# How to Get an NIA Grant

**DOI:** 10.1093/geroni/igaf122.1148

**Published:** 2025-12-31

**Authors:** Kenneth Santora

**Affiliations:** National Institute on Aging, NIH, Bethesda, Maryland, United States

## Abstract

Dr. Santora will provide an overview of the NIA application process, including funding mechanisms and strategies to consider when applying for extramural grants. Training and career development opportunities for early-career investigators at multiple career stages – including undergraduates, advanced degree students, post-docs, and junior faculty – will be featured.

